# Validation of a model of the *GAL* regulatory system via robustness analysis of its bistability characteristics

**DOI:** 10.1186/1752-0509-7-39

**Published:** 2013-05-17

**Authors:** Luca Salerno, Carlo Cosentino, Alessio Merola, Declan G Bates, Francesco Amato

**Affiliations:** 1 Dipartimento di Medicina Sperimentale e Clinica, Università degli Studi Magna Græcia di Catanzaro, Catanzaro, Italy; 2 College of Engineering, Mathematics and Physical Science, University of Exeter, Exeter, UK

**Keywords:** Galactose network, Bistability, Robustness, Domain of attraction, Bifurcation, Local sensitivity, Global sensitivity

## Abstract

**Background:**

In *Saccharomyces cerevisiæ*, structural bistability generates a bimodal expression of the galactose uptake genes (*GAL*) when exposed to low and high glucose concentrations. This indicates that yeast cells can decide between using either the limited amount of glucose or growing on galactose under changing environmental conditions. A crucial requirement for any plausible mechanistic model of this system is that it reproduces the robustness of the bistable response observed *in vivo* against inter-individual parametric variability and fluctuating environmental conditions.

**Results:**

We show how a control-theoretic analysis of the robustness of a model of the *GAL* regulatory network may be used to establish the model’s plausibility in characterizing the persistent memory of different carbon sources, without the need for extensive simulations. Chemical Reaction Network Theory is used to establish that the proposed network model is compatible with structural bistability. The robustness of each of the two operative conditions against fluctuations of the species concentrations is demonstrated by studying the Domains of Attraction of the corresponding equilibrium points. Finally, we use a global robustness analysis method based on Semi-Definite Programming to evaluate the modification of the bistable steady states induced by multiple parametric variations throughout bounded regions of the parameter space.

**Conclusions:**

Our analysis provides convincing evidence for the robustness, and hence plausibility, of the *GAL* regulatory network model. The proposed workflow also demonstrates the power of analytical methods from control theory to provide a direct quantitative characterization of the dynamics of multistable biomolecular regulatory systems without recourse to extensive computer simulations.

## Background

Although yeasts, in common with most cellular organisms, can derive energy from a variety of different molecules, glucose is well-known to be their preferred source, because it provides more energy than any other saccharide. Therefore, yeasts have evolved a complex genetic network to make sure they can consume as much glucose as possible when it is available [1]. In [2], the authors experimentally investigated the regulation of galactose metabolism in *S. Cerevisiæ*, which is mediated by several positive and negative feedback loops acting at the transcriptional level. To probe the system for multistability, two identical cell populations were grown on different media, with and without galactose, respectively. In engineering terms, this amounts to initializing the system at two different operating conditions. Starting from these conditions, the two populations were then exposed to identical galactose concentrations for a period long enough to guarantee the attainment of steady-state conditions. For intermediate levels of the input (galactose concentrations), the two populations settled on different steady states, thus confirming the multistable nature of the system. These and other experimental results have revealed that the *GAL* system exhibits bistable dynamics and that such bistability generates a persistent memory of the type of carbon source consumed by the cell in the past.

In previous work, classical mathematical tools, such as bifurcation analysis, have been used to examine the dynamics of the *GAL* regulatory network, see e.g. [3,4]. Recently, we showed, using a control-theoretic analysis, that the *GAL* network simplified mass-action models proposed in [5,6], do not reproduce the bistable behavior exhibited by the experimental studies of Acar and coworkers; this finding motivated us to propose a new model of the *GAL* system, [7]. In this paper we extend this model and provide a thorough characterization of its dynamical properties, with the aim of validating it as a plausible mechanistic explanation of the persistent memory property. Our approach starts with the analysis of the model’s bistable dynamics as a structural property, arising from the topology of the reaction network. Afterwards, we focus on the study of the robustness of bistability both against fluctuations of the concentrations of the molecular species, caused by endogenous stochastic noise or by exogenous perturbations, and in the face of parametric uncertainties. The principle underpinning these analyses is that the quality of a model cannot be solely evaluated by its capability to reproduce a particular set of experimental measurements. Indeed, a common problem in modeling biological networks is that alternative, structurally different models can fit experimental data equally well [8]. In order to represent a plausible mechanistic description of a biological phenomenon, the model must also replicate an essential feature of biological systems, that is robustness against inter-individual parametric variability and *in vivo* fluctuating environmental conditions [9-12].

Our characterization of the robustness properties of the model starts with an analysis of the Domains of Attractions (DA’s) of the bistable system. Roughly speaking, the DA of an equilibrium point *x*_*e*_ is a region D in the state space, such that $x_{e}\in D$ and every state trajectory crossing D converges asymptotically to *x*_*e*_. DA analysis is crucial for establishing whether the proposed model provides a plausible explanation of the phenomenon under investigation, since the system is actually able to operate around a given equilibrium point with some degree of robustness in the face of both intrinsic stochastic noise and exogenous perturbations only if that equilibrium point possesses a nontrivial DA. Note that the estimation of the DA is, in general, a difficult problem for systems of nontrivial dimension. In our approach we show how, for any mass-action model, it is possible to apply a convex optimization-based method, devised in a purely theoretical context by our group in [13,14], to test whether an assigned polytopic subset of the state space belongs to the DA of an equilibrium point.

We next consider the robustness of the model’s bistable dynamics in the face of uncertain parameter values. Many examples can be found in the literature of studies applying local sensitivity and bifurcations analysis as tools for characterizing the parametric robustness of biological systems, e.g. [15-17]; however, these tools suffer from a significant limitation due to their inability to take into account more than one or two simultaneous parameters variation at the same time. Multi-parametric sensitivity analysis of biomodels is typically performed by resorting to extensive sampling of the admissible parameter space, [18,19], which requires a large large computational effort and can only provide probabilistic conclusions. To overcome these limitations, besides applying local sensitivity and bifurcations analysis, we employ a global sensitivity analysis method proposed in [20,21]. This method is aimed at computing an outer approximation of the region of the state space that contains all the equilibrium points of a given biosystem for all admissible values of the parameters. In our analysis, we devise a straightforward way to adapt this method to provide robustness certificates for bistability in the face of parametric uncertainty.

Thus, beyond our primary goal of validating a new model of the bistable *GAL* regulatory network, we also present what should be a widely applicable and effective procedure for investigating the plausibility of dynamical models of multistable biomolecular circuits, without recourse to large-scale numerical simulations.

## Results

### A new model of the *GAL* regulatory network in *S. Cerevisiæ*

**Figure 1:**
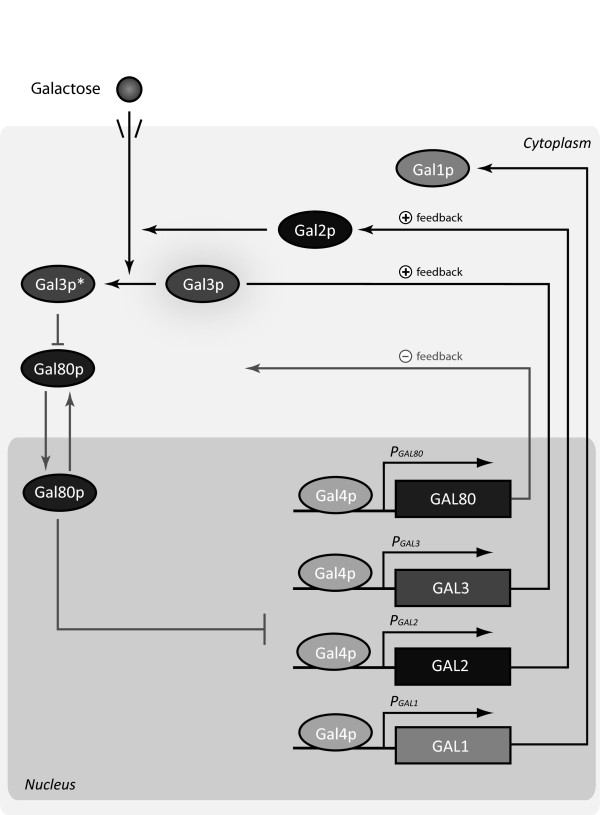
**Schematic diagram of the *GAL *regulatory network in *S. Cerevisiæ*.** Galactose import is guaranteed by Gal2p; internalized galactose activates Gal3p, which sequesters Gal80p in the cytoplasm, shuttling it from the nucleus. The transcriptional activator Gal4p, which is constitutively bound to promoters of the *GAL* genes, is then released from the inhibitory action of Gal80p and activates expression of the *GAL1*, *GAL2*, *GAL3* and *GAL80* genes. The regulatory network features two positive and one negative feedback loops.

The regulatory network of galactose metabolism, depicted in Figure 1, is governed by the following factors: a transcriptional activator protein Gal4p, a signal transducer protein Gal3p and an inhibitor protein Gal80p. In the presence of galactose, Gal4p activates transcription of *GAL2*, *GAL3*, *GAL80*, which are regulatory genes, and of *GAL1* and several other genes (not shown in the figure), which encode the enzymes of the Leloir pathway (the *GAL* genes) of galactose metabolism. The protein encoded by gene *GAL2* acts as a mediator of galactose transport into the yeast cell. In the absence of external galactose, Gal80p binds to the activation domain of Gal4p, thus inhibiting the expression of the *GAL* genes. In the presence of galactose, however, the inducer Gal3p is activated to form the complex Gal80p:Gal3p*, which promotes the shuttling of Gal80p from the nucleus to the cytoplasm. This decreases the fraction of Gal80p-bound Gal4p in the nucleus. Thus, galactose relieves the inactivation of Gal4p and promotes transcription of the *GAL* genes [1].

The mathematical model of this regulatory network considered here is based on mass-action kinetics and represents an extended version of the model proposed in [7]. In the new version of the model, the reaction of reversible dissociation of the complex Gal80p:Gal3p* is explicitly included, since this was found to be essential to ensure robustness of the bistable dynamics, according to the analysis procedure that will be illustrated in the next sections. Moreover, in the new model also the Gal1p protein dynamics are taken into account, since the concentration of this protein is taken as a measure of Gal4p activity in the experiments reported in [2]. model consists of the following set of nonlinear ordinary differential equations (ODE’s),

(1a) $$\begin{array}{ll} & \;{Ġ}_{3}=k_{8}G_{4}-k_{2}G_{3}G_{\text{int}}+k_{r2}G_{3a}-\mu_{13}G_{3}\; \end{array}$$

(1b) $$\begin{array}{ll} & \;{Ġ}_{\text{int}}=k_{1}G_{\text{ex}}G_{2}-k_{2}G_{3}G_{\text{int}}+k_{r2}G_{3a}-\mu_{16}G_{\text{int}}\; \end{array}$$

(1c) $$\begin{array}{lll} & \;{Ġ}_{3a}=k_{2}G_{3}G_{\text{int}}-k_{r2}G_{3a}-k_{4}G_{4,80}G_{3a}-\mu_{3}G_{3a}\; & \;\\ & \;+k_{r4}G_{80,3a}G_{4}-k_{r19}G_{80}G_{3a}+k_{19}G_{80,3a}\; \end{array}$$

(1d) $$\begin{array}{lll} & \;{Ġ}_{4}=k_{5}\;-\;k_{11}G_{4}G_{80}+k_{r11}G_{4,80}+k_{4}G_{4,80}G_{3a}\; & \;\\ & \;-k_{r4}G_{80,3a}G_{4}-\mu_{6}G_{4}\; \end{array}$$

(1e) $$\begin{array}{lll} & \;\;{Ġ}_{80}\;=\;-k_{11}G_{4}G_{80}\;+\;k_{r11}G_{4,80}\;+\;k_{7}G_{4}\;-\;k_{r19}G_{80}G_{3a}\; & \;\\ & \;+k_{19}G_{80,3a}\;-\;\mu_{14}G_{80}\; \end{array}$$

(1f) $$\begin{array}{lll} & \;{Ġ}_{4,80}=k_{11}G_{4}G_{80}-k_{r11}G_{4,80}-k_{4}G_{4,80}G_{3a}\; & \;\\ & \;+k_{r4}G_{80,3a}G_{4}-\mu_{12}G_{4,80}\; \end{array}$$

(1g) $$\begin{array}{lll} & \;{Ġ}_{80,3a}=k_{4}G_{4,80}G_{3a}-k_{r4}G_{80,3a}G_{4}\;-\;\mu_{15}G_{80,3a}\; & \;\\ & \;-k_{19}G_{80,3a}+k_{r19}G_{80}G_{3a}\; \end{array}$$

(1h) $$\begin{array}{ll} & {Ġ}_{2}=k_{9}G_{4}-\mu_{17}G_{2}\; \end{array}$$

(1i) $$\begin{array}{ll} & {Ġ}_{1}=k_{10}G_{4}-\mu_{18}G_{1},\; \end{array}$$

where the description of each state variable is reported in Table 1. The total concentration of external galactose *G*_*ex*_= constant.

**Table 1:** **Steady states of the mass-action model **(1),** with the parameters values given in Table **2

***x***_***e1***_	**Species**	**Description**	***x***_***e2***_
172.8212	*G*_3_	*Gal3p protein*	2711.1839
172.8208	*G*_int_	*internalized galactose*	2711.2003
1.0	*G*_3*a*_	*active Gal3p protein*	318.5443
1.0	*G*_4_	*Gal4p protein*	19.9479
1.0	*G*_80_	*Gal80p protein*	0.3061
21.0604	*G*_4,80_	*Gal4p:Gal80p complex*	2.1126
7.5945	*G*_80,3*a*_	*Gal80p:Gal3p active complex*	589.1342
1.0	*G*_2_	*Gal2p protein*	19.9479
1.0	*G*_1_	*Gal1p protein*	19.9479
1000.0	*G*_*ex*_	*external galactose*	1000.0

Concentration values are all given as [*μM*] units.

**Table 2:** **A set of parameters values that renders system** (1) **bistable**

**Parameter**	**Value**	**Parameter**	**Value**
*k*_1_	0.1814 [ *μM*·h]	*k*_11_	85.8185 [1/h]
*k*_2_	8.4586E-4 [1/ *μM*·h]	*k*_*r*11_	4.7482E-2 [1/ *μM*·h]
*k*_*r*2_	16.6691 [1/h]	*μ*_12_	1.0 [1/h]
*μ*_3_	1.0 [1/h]	*μ*_13_	1.0 [1/h]
*k*_4_	3.0749 [1/ *μM*·h]	*μ*_14_	1.0 [1/h]
*k*_*r*4_	0.1317 [1/h]	*μ*_15_	1.0 [1/h]
*k*_5_	22.0604 [ *μ*M/h]	*μ*_16_	1.0 [1/h]
*μ*_6_	1.0 [1/h]	*μ*_17_	1.0 [1/h]
*k*_7_	29.6549 [1/h]	*μ*_18_	1.0 [1/h]
*k*_8_	181.4157 [1/h]	*k*_19_	36.6342 [1/ *μM*·h]
*k*_9_	1.0 [1/h]	*k*_*r*19_	222.0536 [1/h]
*k*_10_	1.0 [1/h]		

The values have been computed through the CRNT algorithm and then scaled to suitable dimensions (see the results Section ‘Structural analysis of the proposed model’s network topology confirms bistable dynamics’).

The ODE model (1) can be rewritten in compact form as

(2) $$\dot{x}=N\;v\;(x,p),$$

where the species concentrations, namely the state variables listed in Table 1, are denoted by *x*:=(*G*_3_*G*_int_*G*_3*a*_*G*_4_*G*_80_*G*_4,80_*G*_80,3*a*_*G*_2_*G*_1_)^*T*^, the parameters by $p:={\left(k_{1}k_{2}\cdots k_{r19}\right)}^{T}\in {\mathbb{R}}^{23}$ (the full list of parameters is reported in Table 2), $N\in {\mathbb{R}}^{9\times 23}$ is the stoichiometric matrix and $v(x,p)\in {\mathbb{R}}^{23}$ is the vector of reaction rates. Since the parameters are inherently positive, positive values of *x* will result in positive values of *v*(*x*,*p*), i.e. $x\in {\mathbb{R}}_{+}^{9}\Rightarrow v(x,p)\in {\mathbb{R}}_{+}^{23}$. In the next section, we present the results of our analysis of the bistable dynamical properties of this model.

### Structural analysis of the proposed model’s network topology confirms bistable dynamics

The first step of our procedure consists in the analysis of the topology of the regulatory network, to determine whether its structure can admit a bistable behavior. Subsequently, we will determine a possible realization (i.e. a set of parameter values) of model (1) that exhibits bistability.

The persistence of cellular memory exhibited by the galactose regulatory network is a system-level property which results from the interactions of several species in multiple nested feedback loops. Two coupled positive feedback loops involve the galactose permease Gal2p and the signaling protein Gal3p, while a negative feedback loop involves the inhibitor Gal80p. Recall that, according to [22], the existence of a positive-feedback loop, or a mutually inhibitory, double-negative-feedback loop, is a necessary condition for the occurrence of multistability.

Model (2) is said to exhibit bistability if there exist a parameter vector $\bar{p}\in {\mathbb{R}}_{+}^{23}$, and two finite distinct equilibrium points $x_{e1},x_{e2}\in {\mathbb{R}}^{9}$ such that

(3a) $$\begin{array}{l} \text{Nv}(x_{e1},\bar{p})=0\; \end{array}$$

(3b) $$\begin{array}{l} \text{Nv}(x_{e2},\bar{p})=0\; \end{array}$$

The existence of a solution to Eqs. (1b). Such scaling yields an equilibrium concentration of 1mM for *G*_*ex*_; note also that it does not affect other equations, since the kinetic constant *k*_1_ does not appear elsewhere in the model.

### Characterization of the domains of attraction confirms robustness of the bistable equilibria

Subsequently to the determination of the asymptotically stable equilibrium points, a primary goal in the characterization of the behavior of a system is that of estimating the DA’s of such points. Accurate estimates of the DA’s provide valuable information about the ability of a system to reject perturbations driving the system away from its steady state condition. At the same time, the boundaries of the DA’s constitute the concentration thresholds for the activation of the switching mechanism between different operative conditions.

The methodology proposed in [13], which allows to check whether an assigned box in the state space belongs to the DA of an equilibrium, has been employed in our study. It is worth noticing that the main result of [13] leads to a Linear Matrix Inequality (LMI) feasibility problem, which can be solved efficiently via off-the-shelf numerical algorithms.

In order to find the largest possible estimates of the DA’s of *x*_*e*1_ and *x*_*e*2_, namely ${\tilde{D}}_{1}$ and ${\tilde{D}}_{2}$, our procedure takes two small initial polytopic regions, surrounding the equilibrium points, and then iteratively stretches them along the different dimensions of the state space until the feasibility conditions are no longer verified, thus obtaining two inner approximations of the DA’s.

**Figure 2:**
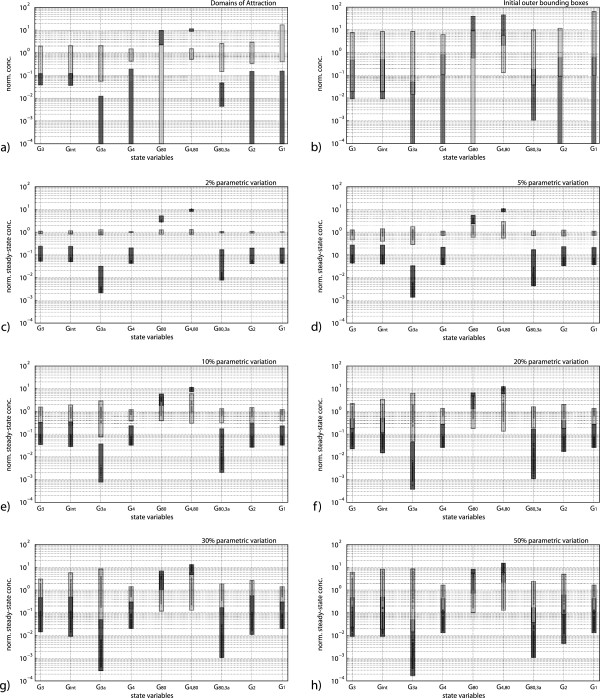
**Estimated regions in the state space.a**) Estimated DA’s of the two equilibrium points; **b**) Initial guess for the computation of the robust steady state subsets; **c**)-**h**) Robust steady state boxes corresponding to several admissible range of simultaneous variation of the parameters *k*_1_,*k*_2_,*k*_5_,*k*_7_,*k*_8_,*k*_9_,*μ*_13_,*μ*_16_,*μ*_17_. In all panels both the low (dark gray) and high (light gray) galactose concentration conditions are considered.

**Figure 3:**
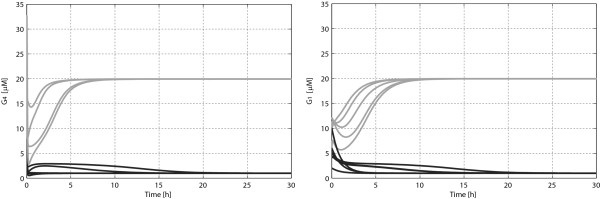
**Free evolutions, for different initial conditions, of the concentrations of Gal4p and Gal1p proteins with the set of parameters given by application of CRNT toolbox.** The curves funnel into low (in dark gray) or high (in light gray), depending on initial conditions, confirming the bistable nature of the system.

The estimates obtained by means of this procedure are

$$\begin{array}{lll} {\tilde{D}}_{1} & =\;[100.82,312.82]\times [100.82,332.82]\times [0.0,4.0]\; & \;\\ & \;\times [0.0,4.0]\times [0.7,3.0]\times [18.06,24.06]\; & \;\\ & \;\times [2.59,27.59]\times [0.0,3.0]\times [0.0,3.0]\; & \;\\ {\tilde{D}}_{2} & =\;[211.18,5213.2]\times [209.20,5622.0]\; & \;\\ & \;\times [18.54,675.5]\times [8.95,30.9]\; & \;\\ & \;\times [0.0,0.7]\times [1.11,3.2]\; & \;\\ & \;\times [89.13,1528.1]\times [6.9479,58.9]\; & \;\\ & \;\times [7.95,320.9]\; & \; \end{array}$$

for *x*_*e*1_ and *x*_*e*2_, respectively (the two boxes are plotted in normalized parallel coordinates in Figure 2a). The validity of these estimates is confirmed by numerical simulations, performed with initial conditions varying within the boxes computed by the proposed approach (see Figure 3). Note that the admissible excursion intervals, as determined by the estimated DA’s (reported in Figure 2a), are fairly large for most of the state variables: looking at Figure 2a one can readily recognize that the key species that drives the switching between the two metabolic conditions is the complex Gal4p:Gal80p, which is associated to a smaller admissible fluctuation interval with respect to the other species (especially in the low galactose concentration condition). Thus, the DA’s analysis highlights that a tight regulation of the concentration of Gal4p:Gal80p is paramount to the proper functioning of the genetic switch.

### Local and global analysis confirm robustness of the bistable equilibria to parametric uncertainty

In this section we provide further support for the plausibility of the proposed model of the *GAL* regulatory system by characterizing its robustness with respect to parametric uncertainties.

The underlying principle is that, in view of the large inter-individual variability of biochemical parameters, for a model to be considered plausible it is not sufficient to reproduce the qualitative behavior of the biological system for just one set of parameter values; instead, this behavior must be exhibited over a nontrivial subset of the parameters space.

First, a classical sensitivity analysis is performed by employing the method discussed in [27]: the state variables ODE’s are coupled to the equations of sensitivity.

This allows us to compute a numerical solution to the whole set of equations, thus simultaneously obtaining both the state variables and the associated sensitivity coefficients.

**Figure 4:**
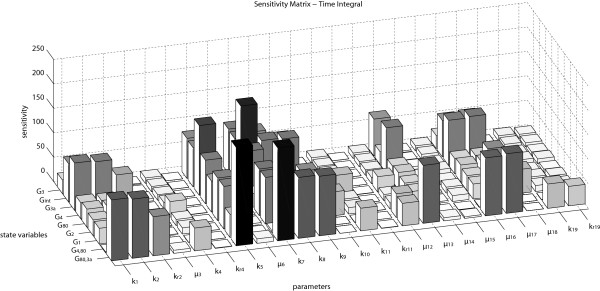
**Local sensitivity analysis at the steady states of the species involved with respect to all model parameters.** The sensitivity matrix denotes the normalized sensitivity coefficients of all species in the model across 20 hours. The sensitivity of the parameters *k*_1_, *k*_2_ (associated in the internalization of external galactose and in activation of Gal3p protein), *k*_5_ (basal expression of transcription factor Gal4p), *k*_7_, *k*_8_, *k*_9_ (associated in the feedback control), *μ*_13_, *μ*_16_ and *μ*_17_ (degradation of Gal3p, internalized galactose and Gal2p proteins, respectively), can more influence the dynamical behavior of this mechanism more than the other parameters.

The normalized sensitivity coefficients for the proposed model are shown in Figure 4: greater sensitivity is exhibited by the parameters involved in the feedback terms (*k*_7_, *k*_8_, *k*_9_), the basal expression of Gal4p (*k*_5_), those involved in the internalization of external galactose and in the activation of Gal3p (*k*_1_, *k*_2_), and the parameters that describe the degradation of Gal3p, internalized galactose and Gal2p (*μ*_13_, *μ*_16_ and *μ*_17_), respectively. It is worth recalling that the indications of robustness provided by the sensitivity coefficients must be taken with caution, keeping in mind that this type of analysis is only valid locally, i.e., in the neighborhood of the nominal values reported in Table 2.

**Figure 5:**
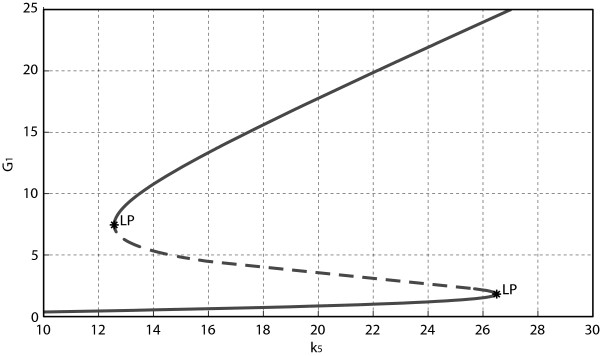
**One-parameter bifurcation diagram.** The diagram has the classical S-shape in the interval [12.57, 26.62], thus the system is bistable for values of *k*_5_ belonging to this interval. (LP, Limit Point).

**Figure 6:**
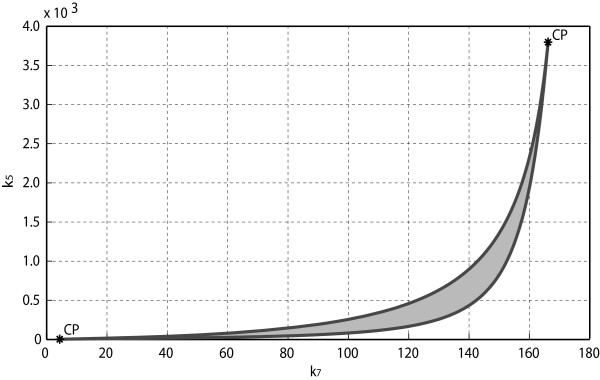
**Two-parameters bifurcation diagram.** Bifurcation curves in the (*k*_7_,*k*_5_)-plane with codimension 2 points are shown. At (*k*_7_,*k*_5_)=(4.363,3.835) and (*k*_7_,*k*_5_)=(166.2,3797.0) two cusp bifurcation points (CP) are detected. The system is bistable for value of (*k*_7_,*k*_5_) included in the shaded area.

We next determine the critical points of the system, i.e., the points at which system’s dynamics undergo abrupt changes. We have conducted a bifurcation analysis with respect to those parameters that exhibit large sensitivity values. Taking Gal1p concentration as the output of our model, the interval of bistability with respect to a certain parameter is delimited by the pair of limit points forming the classical S-shaped bifurcation curve. As an example, the bifurcation diagram generated by variation of *k*_5_ is shown in Figure 5: the admissible range of variation for the parameter *k*_5_ is ([12.57, 26.62]); outside this interval the system loses its bistable behavior. The bifurcation analysis can also be performed by allowing simultaneous variations of two parameters: in this case, the bistability thresholds, corresponding to the limit point bifurcations, are curves in the parameters plane. For example, in Figure 6, where *k*_7_, *k*_5_ have been chosen as bifurcation parameters, we have detected two cusp bifurcation points at (*k*_7_,*k*_5_)=(4.363,3.835) and (*k*_7_,*k*_5_)=(166.2,3797.0). Thus, the shaded region in Figure 6 identifies a set of parameter values within which any value of the pair (*k*_7_,*k*_5_) guarantees bistable behavior (assuming that the other parameters take their nominal values).

Unfortunately, the above methods can efficiently evaluate changes in steady state concentrations only for low-dimensional parameter variations. In [20], a global sensitivity method, named bioSDP, is proposed to evaluate the effect of multiple (simultaneous) parameter variations on the system’s dynamics. More specifically, this method can be used to compute some bounds on the maximum variation of the equilibrium points induced by changes of the parameter values. The computation is based on the solution of a dual problem (see Methods for more details): given a subset of the state space, say *Ω*, the bioSDP algorithm is able to *certificate* that, for any admissible realization of the parameter vector *p*, *Ω* does not contain any equilibrium point.

The bioSDP algorithm takes as inputs the admissible range of variation of the parameters, defined as a box ${ℬ}_{p}$ in the parameter space, and an initial outer approximation, in the form of a box ${\tilde{S}}^{0}$, of the subset $X_{e}$ of all the admissible equilibrium points of system (1) subject to $p\in {ℬ}_{p}$.

Then, these outer boundaries are iteratively narrowed by applying a bisection algorithm. As a result, the state space is partitioned in one or more subsets containing all the equilibrium points that fall inside the initial search subspace ${\tilde{S}}^{0}$. In fact, due to the computational burden, the bisection algorithm can only be applied to systems of dimension less or equal than three (see for example Figure 5 in [21], where a two-dimensional system is analyzed). For higher-order systems like ours, the algorithm resorts to a box shrinkage procedure, i.e., it just tries to reduce the size of the initial box as far as possible.

Due to the above limitations, in our case the method proposed in [20] would not be able to distinguish the two distinct steady state subsets. To overcome this issue, we devise a strategy that leverages the bioSDP algorithm but, instead of computing one set containing all the equilibrium points, aims to separately compute two distinct robust steady state subsets ${\tilde{S}}_{1}$ and ${\tilde{S}}_{2}$, which define the boundaries for the variation of *x*_*e*1_ and *x*_*e*2_, respectively. Thus, we need two initial outer approximation subsets, let us denote them by ${\tilde{S}}_{1}^{0}$ and ${\tilde{S}}_{2}^{0}$, respectively.

Guessing two good initial outer approximations would in general turn out to be a daunting task for systems of nontrivial dimension. In our case, exploiting the previous analysis and by virtue of continuity arguments, we surmise that, for small-enough variations of the parameter values, the DA’s represent good initial guesses. Thus, we let ${\tilde{S}}_{i}^{0}=\rho_{i}{\tilde{D}}_{i}$, *i*=1,2, where *ρ*_*i*_>0 is a scaling factor and apply the bioSDP algorithm separately to these two initial boxes, with *ρ*_*i*_=1; if the algorithm does not find a solution, it is re-applied iteratively with different values of *ρ*_*i*_ in a predefined interval [*ρ*_min_,*ρ*_max_], until a solution is found.

Note that, setting ${\tilde{S}}_{i}^{0}$ as the initial search space for the bioSDP algorithm we are focusing the analysis on those equilibrium points that belong to a neighborhood of *x*_*ei*_, instead of searching for all the equilibrium points. Performing this analysis separately, first on *x*_*e*1_ and then on *x*_*e*2_, enables us to ascertain whether the bistability is preserved against parametric perturbations: the answer is affirmative if we are able to compute two disjoint robust steady state subsets, i.e., ${\tilde{S}}_{1}\cap {\tilde{S}}_{2}=\emptyset$. If this problem is feasible for an assigned parameter box ${ℬ}_{p}$, then we are guaranteed that bistability is preserved for all *p* belonging to ${ℬ}_{p}$.

The initial outer approximations used for our analysis are reported in normalized parallel coordinates in Figure 2b. Their numerical values are

$$\begin{array}{lll} {\tilde{S}}_{1}^{0} & =\;[25.214,1251.211]\times [25.214,1331.199]\; & \;\\ & \;\times [0.0,15.991]\times [0.0,16.0]\times [0.175,12.001]\; & \;\\ & \;\times [4.515,96.242]\times [0.648,110.404]\; & \;\\ & \;\times [0.0,12.001]\times [0.0,12.001],\; & \;\\ {\tilde{S}}_{2}^{0} & =\;[52.898,20852.800]\times [52.326,22664.821]\; & \;\\ & \;\times [4.619,2701.988]\times [2.236,123.599]\; & \;\\ & \;\times [0.0,2.80]\;\times \;[0.278,12.400]\;\times \;[22.269,6112.385]\;\; & \;\\ & \;\times [1.737,235.601]\times [1.987,1283.6].\; & \; \end{array}$$

To alleviate the computational burden of the procedure, the multi-parametric sensitivity analysis has been limited to the parameters subset *Θ*:={*k*_1_,*k*_2_,*k*_5_,*k*_7_,*k*_8_,*k*_9_,*μ*_13_,*μ*_16_,*μ*_17_}, which, according to the local sensitivity analysis, have a major influence on the system dynamics (see Figure 4). The robustness has been evaluated against increasingly larger ranges of parameter variations, corresponding to ±2*%*, ±5*%*, ±10*%*, ±20*%*, ±30*%* and ±50*%*, with respect to the nominal values given in Table 2. Figure 2 displays the computed robust steady state boxes for the various cases. The bistable behavior of the *GAL* regulatory network is guaranteed for parametric variations up to ±20*%* with respect to the nominal parameters value. For such uncertainty values, indeed, the computed subsets ${\tilde{S}}_{1}$ and ${\tilde{S}}_{2}$ are still disjoint, since the intervals of G _3a_ and G _80,3a_ are not overlapping. For parametric variations of ±30*%* or more, the intersection of the two subsets is no longer empty (see Figures 2, panels g and h); in the latter case, it is no longer possible to guarantee that the system preserves bistability for all admissible parameter values.

## Discussion and conclusions

Robustness, intended as the capability to cope with fluctuations of the molecular species concentrations, caused by endogenous and exogenous perturbations, and to preserve biological functions despite inter-individual variability of kinetic parameters, is a key feature of biological systems. This fundamental feature poses an important challenge when trying to describe biological phenomena by means of mechanistic mathematical models: a set of differential/algebraic equations which, for some value of the parameters, interpolates experimental data, cannot be considered a plausible model if it does not possess the aforementioned robustness properties. Recognizing the power of these arguments as tools for testing novel biomodels, and with the aim of supporting the validity of our proposed model of the *GAL* regulatory network, we have devised an analytical procedure which can be exploited to investigate the robustness properties of biomodels of bistable biological systems. The procedure exploits several complementary methods for the analysis of nonlinear quadratic systems (i.e., mass-action models), devised both by our group and by other authors, and its effectiveness has been demonstrated by applying it to thoroughly characterize the robustness of bistability for a new model of the galactose metabolism regulatory system.

The procedure consists of three phases: in the first phase, the properties of the nominal system (i.e., parameters values are assumed to be certain) are investigated, since the first requirement is that the reaction network is structurally compatible with the existence of multiple equilibrium points. This can be ascertained through the use of CRNT, which also allows the computation of a candidate set of parameter values. Subsequently, the second stage of the procedure focuses on the analysis of the DA’s of the equilibrium points, using the method devised in [13], since the DA can be regarded as a robustness measure against perturbations that push the system away from its steady state operative condition. The third phase of the procedure consists in the analysis of the robustness of the system’s bistability with respect to parametric uncertainty. Traditionally, this analysis is based on sensitivity and bifurcations analysis; however, these tools are rather limited, due to their inability to take into account multiple simultaneous parameter variations. To overcome these limitations, we have proposed a multi-parametric robustness analysis strategy: by opportunely leveraging a global sensitivity analysis method, and combining it with the information provided by our DA’s analysis technique, we were able to certify the persistence of bistability in the face of multiple variations of the uncertain parameters.

Beyond its specific application for validation of the proposed model of the *GAL* regulatory network, the overall procedure provides a powerful approach for the analysis and validation of any biochemical network model which is required to robustly reproduce bistable dynamics, underlying persistent memory, molecular switches and cell differentiation phenomena, without recourse to large-scale numerical simulations.

## Methods

### Chemical reaction network theory

Given a reaction network, the capability of the associated model to exhibit two or more equilibrium points depends on the mathematical form of the reaction rates and on the specific values of the kinetic parameters.

While the characterization of multistability for a generic nonlinear system requires an *ad hoc* mathematical treatment, in the case of mass-action systems it can be performed through a powerful analytical tool, namely Chemical Reaction Network Theory (CRNT) [23,24]. CRNT links the structure of a biochemical network, endowed with mass-action kinetics, to the capability of the network to admit multiple positive steady-states. The advantage of CRNT is that it provides an immediate way to analyze the type of dynamical behavior that one can expect from an arbitrarily complex reaction network, just by inspection of the topology of the associated graph. More specifically, CRNT enables us to establish the conditions for the existence, multiplicity and stability of fixed points for the associated ODE system, without even the need to write down the kinetic equations nor to assign values to the kinetic parameters. This point makes CRNT especially suitable for dealing with biomolecular systems, whose parameters are often unknown or subject to significant variability among different individuals.

For a complete description of the CRNT, the interested reader is referred to the original articles [23,24], or to [28] for an introductory overview of the main results. Despite the complexity of its theoretical foundations, the application of the main CRNT results is straightforward through the use of the CRNT algorithm, which is implemented in the CRNT Toolbox^1^. Given the reaction network that we want to study, the algorithm establishes whether the associated mass-action dynamical system can admit multiple positive steady states for some values of the kinetic parameters. In the affirmative case, the algorithm also computes a set of values of the kinetic parameters for which the system is multistable.

### Analysis of the domains of attraction

The exact computation of the DA’s for a nonlinear system is generally a very hard problem to solve, especially for systems of medium/high order. The problem of computing estimates of the DA’s has been studied for a long time and several methods, based on Lyapunov functions, were originally proposed, e.g. in [29,30]. More recently, Chesi has devised novel results concerning DA analysis based on Sum of Squares (SOS) representation of polynomials and Semi-Definite Programming (SDP), [31,32]. Moreover, effective examples of the usefulness of SOS/SDP-based approaches to elucidate the properties of biological systems are provided in [33,34].

Topcu *et al.* in [35,36] have dealt with the topic of estimating a robust DA in the case of uncertain parameters. Compared to the method used in this work, their results can deal with a larger class of systems, namely polynomial dynamical systems; however, they cast the problem in the form of Bilinear Matrix Inequalities (BMI’s), whose solution is much more demanding than LMI’s (used by our approach) and, thus, its practical applicability is limited to systems of low order with few optimization variables..

When dealing with nonlinear quadratic systems, an alternative approach to DA analysis, proposed by Amato and coworkers in [13,14], can be adopted: this method allows one to check whether an assigned box (or, more in general, a polytope) in the state space belongs to the domain of attraction of a given equilibrium point. Such problem can be cast as a LMI feasibility problem [37], which can be effectively tackled through effective off-the-shelf numerical tools. In what follows we provide a brief overview of the main result used in the present work.

First, recall that a box (or, more generally, a polytope) $S\subset {\mathbb{R}}^{N}$ can be described as follows

(4) $$\begin{array}{l} S=\text{conv}\{x_{\left(1\right)},x_{\left(2\right)},\ldots ,x_{\left(r\right)}\}\\ \;=\{x\in {\mathbb{R}}^{N}:{a_{k}}^{T}x\leq 1,k=1,2,\ldots ,q\} \end{array}$$

where *r* and *q* are suitable integers, *x*_(*i*)_ denotes the *i*-th vertex of S, and *conv*[·] indicates the convex hull of the argument. System (2) can be rewritten as

(5) $$\dot{x}(t)=\text{Ax}\;(t)+F(x),$$

with

(6) $$F(x)=\left(\begin{array}{cc} x^{T} & F_{1}\\ x^{T} & F_{2}\\ \vdots \\ x^{T} & F_{n} \end{array}\right)\;x,$$

where $A,F_{i}\in {\mathbb{R}}^{9\times 9}$. We can now precisely state the problem to be solved. Note that, for the sake of brevity, the statement of the problem refers to the zero equilibrium point, i.e. the origin of the state space. Nevertheless, it is easy to generalize the definition and the entire procedure to non-zero equilibrium points, via a change of state variables, as shown in [13].

#### Problem 1

Assume that each eigenvalue of matrix *A* in (5) has strictly negative real part (i.e., the origin is an asymptotically stable equilibrium point); then, given a box S, with the origin of the state space lying in the interior of S, establish whether S belongs to the DA of the zero equilibrium point.

The following Theorem provides sufficient conditions to solve Problem 1.

#### Theorem 1

*The box*S*defined in (4) belongs to the DA of the zero equilibrium of system (5) if there exist scalars γ ∈ (0,1), c >0 and a symmetric positive-definite matrix P such that *

(7a) $$\begin{array}{ll} & \left(\begin{array}{cc} 1 & \gamma {a_{k}}^{T}\\ \gamma a_{k} & P/c \end{array}\right)\geq 0,k=1,2,\ldots ,2n\; \end{array}$$

(7b) $$\begin{array}{cc} & {x_{\left(i\right)}}^{T}(P/c)x_{\left(i\right)}\leq 1,i=1,2,\ldots ,2^{n},\; \end{array}$$

(7c) $$\begin{array}{ll} \;\gamma (A^{T}P+\text{PA})+\left(\begin{array}{c} {x_{\left(i\right)}}^{T}F_{1}\\ {x_{\left(i\right)}}^{T}F_{2}\\ \vdots \\ {x_{\left(i\right)}}^{T}F_{n} \end{array}\right)\;P\;\\ +\;\left(\;\begin{array}{llll} {\;F_{1}}^{T}x_{\left(i\right)} & {F_{2}}^{T}x_{\left(i\right)} & \ldots & {F_{n}}^{T}x_{\left(i\right)} \end{array}\;\right)P\;<\;0,\;i=1,2,\ldots ,2^{n}\; & \; \end{array}$$

For a fixed *γ*, conditions (7) constitute a set of LMI’s, which can be easily solved through off-the-shelf efficient numerical tools (e.g., the LMILAB provided in the MATLAB Robust Control Toolbox [38]).

In order to find the largest possible estimate of the DA, Theorem 1 can be applied iteratively, starting from a small initial box $P_{0}$, surrounding the equilibrium point, and then stretching the box at each iteration along the different dimensions of the state space, until conditions (7) become unfeasible.

### Local sensitivity analysis

Sensitivity analysis unveils to what extent each parameter influences the behavior of a given model and, thus, represents a first evaluation of the model’s robustness. Our sensitivity analysis is conducted according to the method illustrated in [27], which is based on the computation of the sensitivity coefficients: the sensitivity coefficient *s*_*ij*_ is defined as the normalized partial derivative of the state variable *x*_*i*_ with respect to the parameter *p*_*j*_, that is

(8) $$s_{\text{ij}}(x_{i},p_{j},t)=\frac{\partial x_{i}}{\partial p_{j}}\frac{p_{j}}{x_{i}}.$$

The set of differential equations that constitutes the dynamical system is coupled to the equations of the sensitivity coefficients. This allows computing a numerical solution to the whole set of equations, thus simultaneously obtaining both the state variables and the associated sensitivity coefficients.

It is worth recalling that traditional sensitivity analysis methods are only valid locally with respect to a particular point in the model’s parameter space, i.e., in the neighborhood of a certain parameter set. Another significant limitation consists in their capability to consider the sensitivity of the model with respect to the variations of just one parameter at a time; indeed, a model might display low sensitivity to single parameter variations, while being extremely sensitive to simultaneous multiple parameter changes.

### Bifurcations analysis

Bifurcation analysis is concerned with the study of how parameter variations affect the number, type and location of attractors, e.g., equilibrium points of a dynamical system. Let us consider a generic nonlinear system, with state variables denoted by *x*, and depending on a parameters vector *p*,

(9) $$\dot{x}(t)=f(x(t),p).$$

A bifurcation occurs at values of *p* such that small changes of the parameters can dramatically alter the number or types of attractors of system (9) [39].

The changes in the map of equilibrium points can be effectively visualized by using a ’bifurcation diagram’, in which the steady state value of one state variable is plotted as a function of a bifurcation parameter. The calculation of bifurcations can be performed through continuation softwares, like the MatCont package [40], which we have used to perform the analysis illustrated in Figures 5 and 6.

Bifurcation diagrams are powerful tools in order to investigate the robustness of nonlinear biomodels in the face of parametric uncertainty. However, it is necessary to take into account that a) analytical solutions for bifurcations are only available for low-dimensional models, and b) that bifurcation diagrams are practically applicable only to study the effect of one or two parameters variation at a time.

### Global sensitivity analysis via infeasibility certificates

In view of the limitations of the approaches presented in the previous two sections, while it is possible to employ them to obtain preliminary information on the parametric robustness of a given model, particular care must be taken in drawing any conclusions about global properties of the system under investigation.

To overcome these limitations, we have exploited a global sensitivity analysis technique for biochemical networks proposed in [20]. Given the admissible parameters variation box, the approach proposed by Waldherr et al. allows one to compute an outer approximation, $\tilde{S}$, of the region of the state space that contains all the equilibrium points, denoted by $X_{e}$. The problem can be formalized as follows.

#### Problem 2

*Given system (2) and a box*${ℬ}_{p}$*in the parameter space, compute a box*$\tilde{S}$*such that*$\tilde{S}⊇X_{e}$,*where*

(10) $$X_{e}=\{x\in {\mathbb{R}}^{n}|\exists p\in {ℬ}_{p}:N\;v(x,p)=0\}.$$

Note that, apart from trivial cases, the calculation of an analytical form of $X_{e}$ is practically impossible. Moreover, computational brute-force approaches are applicable only to very low-order systems. Monte-Carlo techniques can be applied in the other cases, although they may require a large computational effort and guarantee only probabilistic results.

Problem 2 can be effectively solved via the method proposed by Waldherr et al., which can be formulated in the form of the following feasibility problem

(11) $$\begin{array}{c} \text{find}\;x\in {\mathbb{R}}^{n},p\in {\mathbb{R}}^{m}\\ \text{s.t.}\;\text{Nv}(x,p)=0\\ {\;p}_{j,\text{min}}\leq p_{j}\leq p_{j,\text{max}},\;j=1,\ldots m\\ {\;x}_{i,\text{min}}\leq x_{i}\leq x_{i,\text{max}},\;i=1,\ldots n, \end{array}$$

where *p*_*j*,min_, *p*_*j*,max_, *j*=1,…,*m*, define the admissible parameter box ${ℬ}_{p}$, and *x*_*i*,min_, *x*_*i*,max_, *i*=1,…,*n*, are the extremal values of the box $\tilde{S}$ of the state space to be tested as a candidate solution to Problem 2.

The optimization problem (11) is not easy to deal with from the computational point of view. However, it can be tackled by solving its dual version, that is the problem of computing regions of the state space that are guaranteed not to contain any steady state for any parameter value in ${ℬ}_{p}$. The latter can be relaxed to become a SDP problem [41], and solved by means of computationally efficient convex optimization tools. For a detailed description of this procedure, the reader is referred to [20,21]. In this works, the computation of a solution to problem 2 constitutes the core of an iterative procedure, implemented by the bioSDP algorithm: starting from an initial large region of the state space, the algorithm tries to compute one or more partitions containing $X_{e}$. The procedure is very effective for low-order systems (*n*≤3), since in this case a bisection algorithm can be used for the partitioning. For systems of higher order, a box shrinkage procedure is employed, which can only return one partition $\tilde{S}$ and, therefore, is not useful for analyzing the persistence of bistability.

In order to solve this problem, we have devised an alternative strategy, which combines the results of the DA’s analysis with the bioSDP algorithm and has proven to be effective in the analysis of our case study. The details of this approach have been already reported in the results Section ‘Local and global analysis confirm robustness of the bistable equilibria to parametric uncertainty’.

## Endnotes

^1^The CRNT algorithm is implemented in the CRNT toolbox, which is freely available at http://www.che.eng.ohio-state.edu/~feinberg/crnt/

## Competing interests

The authors declare that they have no competing interests.

## Authors’ contributions

CC and LS conceived the idea, designed the study and generated the GAL model. CC, AM, DGB and FA developed the methodological pipeline for robust analysis of bistability. LS performed the numerical experiments. CC and LS drafted the first version of the manuscript. All authors contributed to the writing of, read and approved the final manuscript.
